# Response of soil nutrient dynamics and stoichiometric characteristics in blueberry to fertilization rates

**DOI:** 10.1371/journal.pone.0335223

**Published:** 2025-11-11

**Authors:** Xianyong Wang, Xiaoli An, Na Lei, Delu Wang

**Affiliations:** College of Forestry, Guizhou University, Guiyang, Guizhou, China; National Institute of Agricultural Research - INRA, MOROCCO

## Abstract

To accurately understand the fertilizer requirements of blueberries during various growth stages, this study utilized 7-year-old rabbiteye blueberry cultivar ‘Powderblue’ as the research material. Based on leaf ecological stoichiometry, combined with photosynthetic rate and leaf area, the theoretical nitrogen (N), phosphorus (P), and potassium (K) requirements were calculated every 10 days across four growth stages: flowering (S1), fruit setting (S2), young fruit development (S3), and fruit expansion (S4). Fertilization experiments with 1× (1F), 5× (5F), 10× (10F), and 15× (15F) of the theoretical nutrient demands were conducted during these four stages to investigate the effects of varying NPK application rates on soil nutrient content throughout the annual growth cycle. Results indicated that high fertilizer treatments significantly increased short-term soil N, P, and K availability; however, their concentrations decreased by day 10 post-application, indicating the necessity for topdressing at 10-day intervals to maintain nutrient effectiveness. Soil organic carbon and organic matter levels tended to increase on day 5 post-fertilization but generally decreased by day 10, with more pronounced declines observed in low-rate treatments (e.g., 1F). Soil stoichiometric ratios remained relatively stable across fertilizer gradients, suggesting that plants absorbed nutrients proportionally while maintaining a balance of residual nutrients. Blueberries exhibited higher N uptake compared to P and K across all stages, with the fruit expansion stage demonstrating the highest nutrient absorption rates—2.95 to 10.55 times (N), 3.05 to 6.53 times (P), and 2.77 to 8.54 times (K) those of the preceding stages. These findings underscore the necessity of prioritizing nitrogen (N) supply while dynamically adjusting phosphorus (P) and potassium (K) application ratios during each growth phase, particularly during the fruit expansion stage. Furthermore, balancing soil organic matter mineralization and accumulation is crucial for achieving synergistic regulation of nutrient use efficiency and soil health.

## 1. Introduction

Ecological stoichiometry is a discipline that focuses on the balance relationships among multiple chemical elements in ecosystems, playing a crucial role in the study of nutrient cycling among plants, microorganisms, and soil. It has been recognized as an important method for investigating nutrient cycling and dynamic changes in terrestrial ecosystems [1–3]. Soil carbon (C), nitrogen (N), phosphorus (P), and potassium (K) are essential chemical nutrients for plant growth and development, performing vital functions throughout the growth process [4,5]. Specifically, soil organic carbon (SOC) represents the largest carbon pool in terrestrial ecosystems and plays a critical role in global carbon cycling [6]. Soil nitrogen (N) serves as the primary component of proteins, which are closely associated with crop yield and quality while being the most dynamic element affecting soil fertility [7]. Soil phosphorus (P) is involved in the synthesis of energy substances such as adenosine triphosphate and reduced coenzymes in plants, indirectly facilitating the conversion of other elements and energy cycles [8]. Soil potassium (K) plays a crucial role in promoting photosynthesis, protein synthesis, and intracellular enzyme activation, thereby maintaining intracellular ion balance and regulating stomatal movement [9]. Within the soil system, stoichiometric relationships among carbon (C), nitrogen (N), phosphorus (P), and potassium (K) exhibit homeostasis, influencing soil microbial dynamics, plant root nutrient uptake, and nutrient cycling processes, which are essential for maintaining the structure, function, and stability of the entire soil system [10]. In a study examining the effects of nitrogen and phosphorus addition on soil nutrient content and the ecological stoichiometric characteristics of desert steppe, it was found that the desert steppe maintains a relatively stable C:N ratio, while the N:P ratio is primarily limited by soil phosphorus content [11]. Additionally, it was observed that as the age of Chinese fir forests increases, the soil C:N ratio remains unchanged, whereas the C:P and N:P ratios reach their maximum in 24-year-old mature forest [12]. Furthermore, altitude, slope, soil bulk density, soil water content and canopy density are identified as the main factors affecting the ecological stoichiometric characteristics of rubber forest soil [13]. Moreover, the contents and ratios of C, N, P, and K in the soil directly influence plant nutrient absorption and utilization, potentially altering biomass allocation and ecological strategies [14]. Therefore, studying the contents of soil C, N, P, and K and their ecological stoichiometric characteristics is critical for revealing the influencing factors and mechanisms underlying ecosystem structure, processes, and functions.

Blueberries (*Vaccinium* spp.), commonly referred to as bilberries or blueberries, belong to the Ericaceae family and are classified as perennial deciduous or evergreen shrubs [15]. These fruits are abundant in anthocyanins and offer numerous health benefits, including antihypertensive and lipid-lowering effects [16], anticancer properties [17], eye protection anti-inflammatory functions [18], and preventive effects against cardiovascular and neurodegenerative diseases [19,20]. As a result, they have been recognized by the United Nations Food and Agriculture Organization as one of the world’s five healthiest fruits, earning the titles “Queen of Fruits” and “King of Berries” [21,22]. In China, however, irrational fertilization practices prevail in blueberry cultivation, with growers relying excessively on empirical methods. Many mistakenly believe that increased fertilizer inputs directly correlate with higher yields, leading to over-fertilization, which can cause soil salinization, inhibit plant growth, and even result in mortality [23]. While blueberries require substantial nutrients during critical growth stages such as flowering and fruiting, their specific fertilizer demands at various developmental phases remain poorly understood. Therefore, this study conducts fertilization experiments grounded in ecological stoichiometry, aligned with the actual physiological requirements of blueberries, aiming to provide a theoretical foundation for scientific fertilization practices in blueberry production.

## 2. Materials and methods

### 2.1. Experimental materials

The experiment was conducted at the experimental base of Guizhou University, located in Guiyang City, Guizhou Province (106°27′-106°52′E, 26°11′-26°34′N). The test material consisted of 7-year-old rabbiteye blueberry (Vaccinium ashei Reade) cultivar ‘Powderblue’, with an average plant height of 84.26 cm, a crown width of 64.39 cm × 51.62 cm, and ground diameter of 12.09 mm. On December 23, 2022, the plants were transplanted into reinforced planting bags (top diameter 30 cm, bottom diameter 30 cm, height 25 cm), with one plant per bag. The cultivation substrate used was a low-nutrient acidic universal medium, composed of peat, quartz sand, and perlite in a volume ratio of 3:1:1. This substrate was characterized by total nitrogen (TN) of 0.58%, total phosphorus (TP) of 0.64 g/kg, total potassium (TK) of 4.61 g/kg, available nitrogen (AN) of 72.03 mg/kg, available phosphorus (AP) of 16.28 mg/kg, available potassium (AK) of 37.74 mg/kg, soil organic matter (SOM) of 501.97 g/kg, and a pH of 4.97.

### 2.2. Experimental design

Critical nutrient levels for rabbiteye blueberry (Vaccinium ashei) leaves have been established in the literature as follows: nitrogen (N) at 1.20% to 1.70%, phosphorus (P) at 0.08% to 0.17%, and potassium (K) at 0.28% to 0.60% [24,25]. Utilizing a leaf carbon (C) concentration of 45% as a reference [26,27], ecological stoichiometric ratios of rabbiteye blueberry leaves were calculated (see Table 1). The photosynthetic rate was measured using a Li-6800 portable photosynthetic apparatus (Beijing Ligaotai Technology Co., Ltd.) and the photosynthetic area was assessed with a YMJ series handheld leaf area meter (Shandong Holder Electronic Technology Co., Ltd.) across four developmental stages: flowering stage (S1), fruit setting stage (S2), young fruit stage (S3) and fruit expansion stage (S4) (refer to Table 2). Daily carbon production was calculated using the formula: total photosynthetic yield = photosynthetic rate × leaf area × photosynthetic duration [28]. Theoretical nutrient demands for each developmental stage were derived by integrating leaf stoichiometric ratios and subsequently converted to fertilizer requirements (see Table 3). The photosynthetic rate was averaged over 2–3 consecutive days, leaf area represented the average per plant, and the photosynthetic duration was standardized at 8 hours per day.

**Table 1:** Presents the range of ecological stoichiometric ratios for rabbiteye blueberry leaves.

Stoichiometric ratio	Range	Mean value
C:N	26.47 ~ 37.50	31.03
C:P	264.71 ~ 562.50	360.00
C:K	75.00 ~ 160.71	102.27
N:P	7.06 ~ 21.25	14.15
N:K	2.00 ~ 6.07	4.04
K:P	1.65 ~ 7.50	4.57

Note: The ranges of C:N, C:P and C:K in the table are derived from the “maximum and minimum values of carbon/ nutrient elements”, while the average value is derived from the “carbon/ nutrition”.

**Table 2:** Presents the calculation parameters for fertilizer application at each growth stage.

Fertilizer time	Gross photosynthetic rate (µmol m⁻^2^ s⁻^1^)	Average leaf area per plant (cm^2^)
S1-1 (2023.3.25)	7.65	517.53
S1-2 (2023.4.5)	5.97	775.00
S2-1 (2023.4.16)	7.30	1325.82
S3-1 (2023.4.26)	9.27	1452.10
S4-1 (2023.5.13)	9.43	1481.20
S4-2 (2023.5.23)	10.21	1544.31
S4-3 (2023.6.2)	10.95	1485.73
S4-4 (2023.6.12)	11.81	1795.96

Note: Sn-m, where n represents the developmental periods 1–4, and m indicates the mth fertilization.

**Table 3:** Details the amount of fertilizer applied to blueberries over a 10-day period during each stage (Compounds).

Fertilizer time	Varieties of fertilizer	Fertilization rate (g/ plant)			
		1F	5F	10F	15F
S1-1 (2023.3.25)	N fertilizer	0.2080	1.0401	2.0802	3.1204
	P fertilizer	0.0154	0.0772	0.1545	0.2317
	K fertilizer	0.0299	0.1494	0.2988	0.4482
S1-2 (2023.4.5)	N fertilizer	0.2431	1.2154	2.4308	3.6463
	P fertilizer	0.0180	0.0902	0.1805	0.2707
	K fertilizer	0.0349	0.1746	0.3492	0.5237
S2-1 (2023.4.16)	N fertilizer	0.5090	2.5452	5.0903	7.6355
	P fertilizer	0.0378	0.1890	0.3780	0.5669
	K fertilizer	0.0731	0.3656	0.7311	1.0967
S3-1 (2023.4.26)	N fertilizer	0.7075	3.5374	7.0749	10.6123
	P fertilizer	0.0525	0.2627	0.5253	0.7880
	K fertilizer	0.1016	0.5081	1.0162	1.5243
S4-1 (2023.5.13)	N fertilizer	0.7340	3.6701	7.3402	11.0103
	P fertilizer	0.0545	0.2725	0.5450	0.8175
	K fertilizer	0.1054	0.5272	1.0543	1.5815
S4-2 (2023.5.23)	N fertilizer	0.8289	4.1446	8.2891	12.4337
	P fertilizer	0.0615	0.3077	0.6155	0.9232
	K fertilizer	0.1191	0.5953	1.1906	1.7859
S4-3 (2023.6.2)	N fertilizer	0.8550	4.2748	8.5495	12.8243
	P fertilizer	0.0635	0.3174	0.6348	0.9522
	K fertilizer	0.1228	0.6140	1.2280	1.8420
S4-4 (2023.6.12)	N fertilizer	1.1147	5.5737	11.1474	16.7210
	P fertilizer	0.0828	0.4138	0.8277	1.2415
	K fertilizer	0.1601	0.8006	1.6012	2.4017

Note: Nitrogen fertilizer is represented by ammonium sulfate ((NH_4_)_2_SO_4_, AR, ≥ 99%), phosphorus fertilizer by calcium superphosphate (Ca(H_2_PO_4_)_2_·H_2_O, AR, ≥ 92%), and potassium fertilizer by potassium sulfate (K_2_SO_4_, AR, ≥ 99%).

Due to the uncertainty surrounding the absorption and utilization efficiency of fertilizer by blueberry plants as well as the effective conversion efficiency of applied fertilizers in the soil, a discrepancy exists between the theoretical and actual fertilizer requirements. Consequently, it is essential to conduct gradient fertilization tests to verify these requirement. In this study, four fertilizer gradients were established: 1× (1F), 5× (5F), 10× (10F), and 15× (15F) of the 10-day theoretical requirement, applied independently across four developmental stages (with no cross-treatment). This resulted in a total of 16 treatments (4 stages × 4 gradients), each replicated three times (7 plants per replicate), culminating in 336 plants. Fertilizers (chemical compounds) were dissolved in 700 mL of water and applied via slow irrigation. The test plants were randomly placed in a plastic film greenhouse for cultivation. The arrangement of plants for the four treatments was organized in a 12 × 7 layout, consisting of 12 columns with 7 plants in each column. With the exception of the experimental treatment, all other management measures were standardized. The definitions of the developmental stages are as follows:

Flowering (S1): 5% to 90% flower bloom;
Fruit setting (S2): 5% to 90% fruit set;
Young fruit (S3): Post-90% fruit set to the initiation of rapid expansion in 5% of the fruit;
Fruit expansion (S4): From the initiation of rapid expansion in 5% of the fruit to 5% fruit maturity.

### 2.3. Sample collection and determination

**Fig 1:**
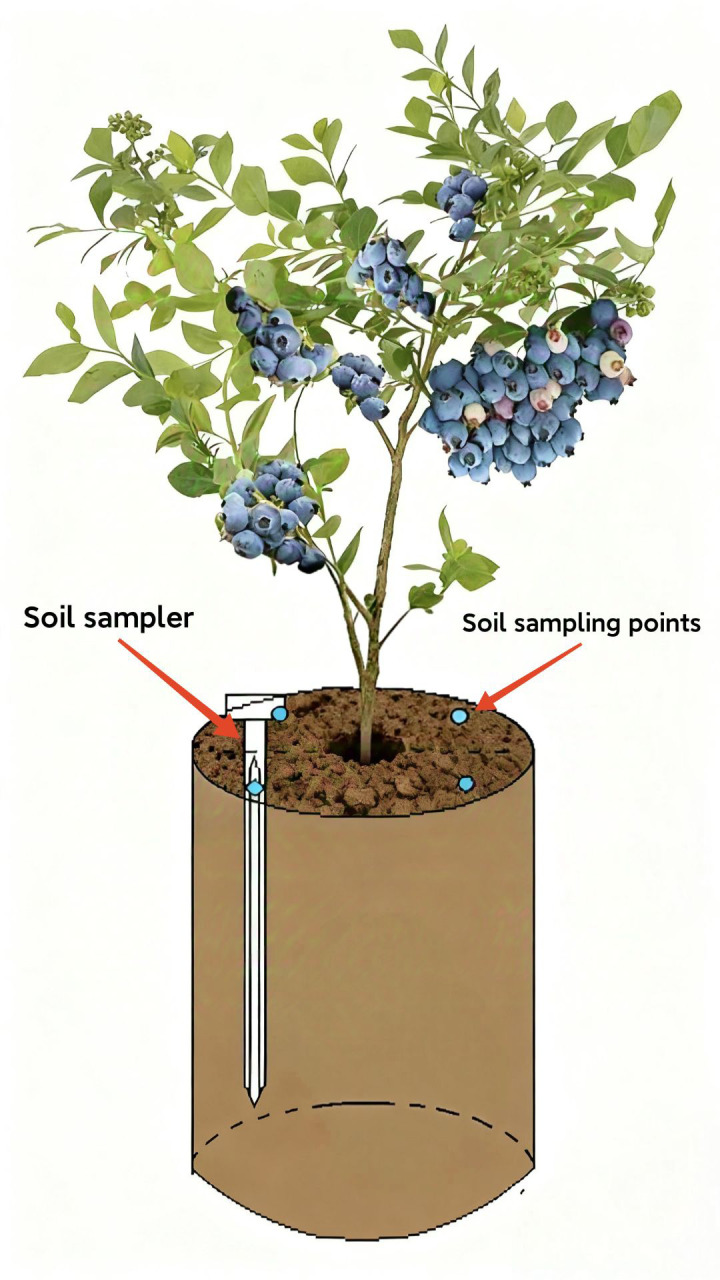
Schematic diagram of soil sample collection.

Soil samples were collected the day before each fertilization and on the 5th and 10th days following each fertilization. A soil sampler (total length of 35 cm, diameter of 10 mm) was used to collect samples, with four points taken from each pot of seedlings (see Fig 1). Three biological replicates were obtained for each treatment, with 100 g of soil collected for each replicate.

Soil organic carbon (SOC) and organic matter (SOM) contents were determined using the oil bath-heated potassium dichromate oxidation method. Total nitrogen (TN) was measured through sulfuric acid-perchloric acid digestion, followed by alkaline hydrolysis diffusion and semi-micro Kjeldahl analysis. Total phosphorus (TP) was quantified using the molybdenum-antimony colorimetric method, while total potassium (TK) was analyzed via flame photometry. Available nitrogen (AN) was determined through alkaline hydrolysis diffusion combined with semi-micro Kjeldahl analysis. Available phosphorus (AP) was measured using sodium bicarbonate extraction and molybdenum-antimony colorimetry, and available potassium (AK) was analyzed via ammonium acetate extraction and flame photometry. Nutrient uptake calculations were performed as follows: Nutrient uptake per plant during a stage = Initial soil nutrient content at the stage’s start + Fertilizer nutrient input during the stage – Final soil nutrient content at the stage’s end; Daily nutrient uptake rate = Total nutrient uptake per plant/ Number of days in the stage.

### 2.4. Data processing

Data sorting, calculations, and mapping were conducted using Excel 2019 and Origin 2024. One-way analysis of variance (ANOVA) was performed on the data utilizing SPSS version 19.0. The Least Significant Difference (LSD) method was employed to compare differences among various data groups. A significance level of α = 0.05 was established, and statistical differences were indicated using the sequential letter labeling method.

## 3. Results and analysis

### 3.1. Effect of fertilizer application rate on soil nitrogen content

**Fig 2:**
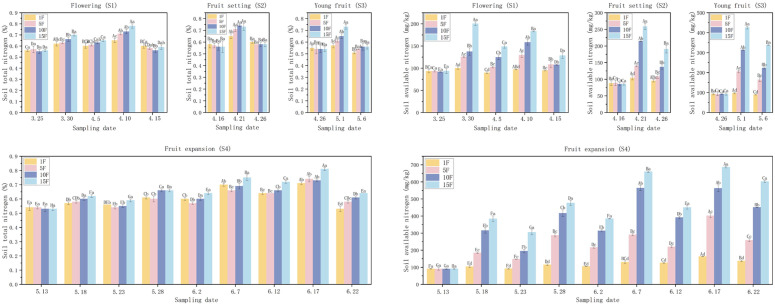
Effects of different fertilization rates on soil TN and AN contents in different periods. Note: Different uppercase letters in the figure indicate that the differences between the same treatment at different times are significant, while different lowercase letters denote that the differences between different treatments at the same time are significant (*P* < 0.05).

As demonstrated in Fig 2, independent fertilization at each growth stage significantly influenced the contents of total nitrogen (TN) and alkaline hydrolyzable nitrogen (AN) in blueberry plants. Following each fertilization during the flowering stage, both TN and AN contents exhibited an increase on day 5, followed by a decrease on day 10 across all fertilizer gradients. Specifically, on day 5 after the initial fertilization, TN contents rose by 10.36% to 25.20% and AN by 7.37% to 113.99% compared to pre-fertilization levels, with the 15F treatment yielding the hightest increases. After the second fertilization, TN increased by 9.45% to 19.23% and AN by 8.36% to 27.13% on day 5, with the most significant TN increase observed in the 15F treatment and the largest AN increase noted in the 10F treatment.

During the fruit setting stage, total nitrogen (TN) contents increased by 12.99%, 24.15%, 30.93%, and 30.27% in the 1F to 15F treatments respectively on day 5 post-fertilization. This was followed by decreases of 8.34%, 17.21%, 22.16%, and 21.42% on day 10. Similarly, amino nitrogen (AN) contents rose by 18.27%, 57.63%, 151.02%, and 202.57% on day 5, then declined by 8.3%, 22.50%, 36.24%, and 26.37% on day 10. After fertilization during the young fruit stage, TN contents increased by 3.74% to 34.22%, while AN increased by 4.43% to 361.28% on day 5. Although both nutrients decreased from day 5 to day 10, most post-decline values remained higher than pre-fertilization levels, with the exception of TN and AN in the 1F treatment, and TN in the 5F treatment. During the fruit expansion stage, TN and AN contents exhibited a consistent pattern of increases on day 5 and decreases on day 10 across all four fertilization treatments. Fig 1 also illustrates cumulative increases in TN and AN from May 18 to June 17, with higher increments observed in treatments receiving more frequent fertilization. Compared to pre-fertilization baselines, day 5 TN increases ranged from 6.53% to 16.44%, 9.42% to 18.96%, 14.31% to 18.25%, and 10.53% to 15.17% across fertilizer gradients, while AN increases spanned from 14.41% to 321.51%, 24.82% to 114.02%, 23.88% to 79.75%, and 29.49% to 82.04%. Notably, AN demonstrated greater proportional increases relative to TN across all treatments.

### 3.2. Effect of fertilizer application rate on soil phosphorus content

**Fig 3:**
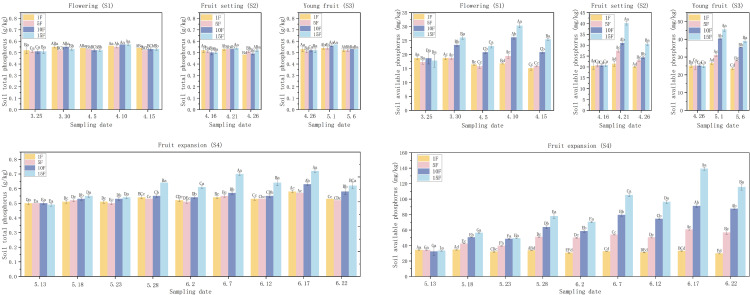
Effects of different fertilization rates on soil TP and AP contents in different periods. Note: Different uppercase letters in the figure indicate that the differences between the same treatment at different times are significant, while different lowercase letters denote that the differences between different treatments at the same time are significant (*P* < 0.05).

As shown in Fig 3, during the flowering stage, the total phosphorus (TP) content in the soil remained relatively stable, ranging from 0.51 to 0.57 g/kg, irrespective of the timing of fertilization. The available phosphorus (AP) exhibited increases of 0.04% to 46.61% and 2.69% to 31.97% on day 5 following the first and second fertilizations respectively; however, it decreased by day 10 while still generally exceeding pre-fertilization levels. Notable exceptions included the AP levels in the 1F and 5F treatments on April 5, as well as the 1F treatment on April 15, indicating an insufficient phosphorus supply to meet the requirements over the 10-day period.

During the fruit-setting stage, TP increased by 2.57% to 8.16% on day 5 post-fertilization. By day 10, the TP levels in the 1F, 5F, and 10F treatments fell below pre-fertilization levels, while the 15F treatment maintained a higher TP content. The AP increased by 5.13% to 93.01% on day 5 (with the maximum observed in the 15F treatment), but decreased by day 10 with only the 1F treatment falling below baseline levels. Following fertilization during the young fruit stage, TP increased by 3.40% to 10.55% on day 5. By day 10, TP levels in the 5F, 10F, and 15F treatments remained 2.05%, 2.26%, and 3.48% above pre-fertilization levels, while the 1F treatment decreased by 1.30%. The AP increased rapidly by 5.94%, 24.01%, 61.03%, and 82.46% across the 1F to 15F treatments on day 5, before declining by 10.00% to 14.37% on day 10. Only the AP in the 1F treatment fell 6.01% below baseline, while the other treatments remained elevated. During the fruit expansion stage, both TP and AP contents exhibited significant treatment × time interactions. Across the four fertilization treatments, both nutrients demonstrated a consistent pattern similar to nitrogen: increases on day 5 followed by decreases on day 10, highlighting the necessity of phosphorus topdressing at 10-day intervals to maintain nutrient availability. In contrast to nitrogen, the increases in TP and AP on day 5 (ranging from 2.46% to 17.85% and 0.94% to 69.12%, respectively) were smaller in magnitude compared to total nitrogen (TN) and ammonium nitrogen (AN).

### 3.3. Effect of fertilizer application rate on soil potassium content

**Fig 4:**
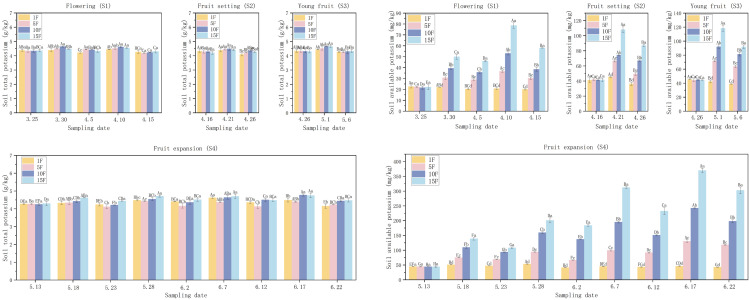
Effects of different fertilization rates on soil TK and AK contents in different periods. Note: Different uppercase letters in the figure indicate that the differences between the same treatment at different times are significant, while different lowercase letters denote that the differences between different treatments at the same time are significant (*P* < 0.05).

As illustrated in Fig 4, the application of varying amounts of nitrogen, phosphorus, and potassium fertilizers resulted in significant differences in both total potassium and available potassium content in the soil, attributable to the differing fertilization rates. Furthermore, the trends observed in total potassium and available potassium content following the four fertilization treatments were consistent with those of nitrogen and phosphorus.

### 3.4. Effect of fertilization on soil organic carbon and organic matter content

**Fig 5:**
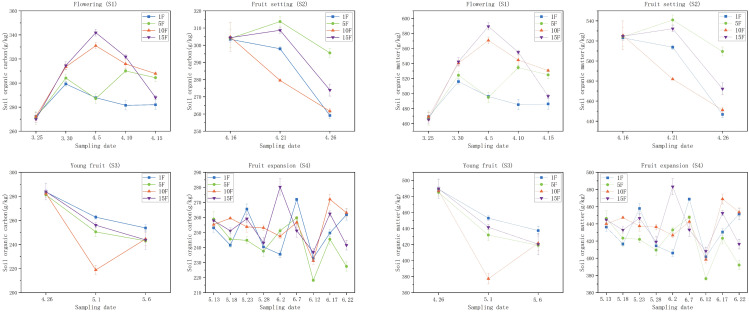
Effects of different fertilization rates on soil SOC and SOM contents in different periods. Note: Different uppercase letters in the figure indicate that the differences between the same treatment at different times are significant, while different lowercase letters denote that the differences between different treatments at the same time are significant (*P* < 0.05).

As illustrated in Fig 5, soil organic carbon (SOC) and organic matter (SOM) demonstrated consistent trends across various fertilization treatments throughout each growth stage. During the flowering stage, SOC and SOM increased in all treatments on day 5 following the initial fertilization. By day 10, treatments 10F and 15F continued to exhibit increases, while treatments 1F and 5F experienced a decline from their day 5 levels. Following the second fertilization, both SOC and SOM decreased in the 1F, 10F, and 15F treatments on days 5 and 10, whereas treatment 5F showed an increase on day 5, followed by a decrease on day 10.

During the fruit setting stage, SOC increased by 3.21% and 1.42% in the 5F and 15F treatments on day 5, respectively, but decreased by 1.80% and 8.31% in the 1F and 10F treatments. By day 10, all treatments exhibited SOC levels below pre-fertilization levels. SOM mirrored the trends observed in SOC on both days 5 and 10. After fertilization during the young fruit stage, SOC and SOM declined in the 1F, 5F, and 15F treatments on both days 5 and 10, with treatment 5F showing a decrease on day 5 followed by an increase on day 10. By day 10, SOC and SOM had dropped by 10.55% to 14.15% compared to pre-fertilization levels. During the fruit expansion stage, following four fertilizations, SOC and SOM decreased on day 5 after the first two fertilizations, increased after the last two fertilizations, and then decreased again by day 10. All treatments reached their minimum SOC and SOM levels on June 12, which was day 10 after the third fertilization.

### 3.5. Effect of fertilizer application rate on soil stoichiometric ratio

As shown in Table 4, independent fertilization during each growth stage exerted significant effects on soil stoichiometric ratios. However, numerical fluctuations among treatments within the same stage remained minimal, indicating that stoichiometry-based fertilization maintained nutrient proportions within stable ranges. During the flowering stage, the soil C:N, C:P, and C:K ratios in the 5F and 10F treatments were significantly higher than those in other gradients. The N:P (10.63 to 11.30), N:K (1.33 to 1.40), and K:P (7.82 to 8.17) ratios showed no significant differences across treatments. After fruit setting fertilization, the 5F treatment achieved the highest C:N (50.59), C:P (583.49), and C:K (69.81) ratios, significantly exceeding those of other treatments. The maximum N:P, N:K, and K:P ratios were observed in the 1F, 1F, and 10F treatments respectively, closely related to the pronounced SOC enhancement in the 5F treatment. During the young fruit and fruit expansion stages, the 1F treatment exhibited the highest C:N, C:P, and C:K ratios. This was a result of reduced fertilizer inputs that slowed SOC mineralization and decomposition, combined with lower residual N/P/K after plant uptake. Across these stages, the N:P (9.90 to 10.51; 9.97 to 11.10), N:K (1.21 to 1.29; 1.29 to 1.44), and K:P (7.9 to 8.21; 7.16 to 8.08) ratios remained within reasonable ranges, indicating stable stoichiometry despite varying fertilizer rates. This suggests that plants absorbed nutrients proportionally, maintaining consistent residual nutrient ratios.

**Table 4:** Effects of different fertilizer application rates on soil stoichiometric ratio characteristics at different stages.

Treatment		C:N	C:P	C:K	N:P	N:K	K:P
Fertilizing season	Fertilizer amount						
S1	1F	47.33 ± 0.56c	519.46 ± 4.37c	66.04 ± 0.60b	10.98 ± 0.22ab	1.40 ± 0.01a	7.82 ± 0.05c
	5F	52.86 ± 0.52b	584.57 ± 3.59a	72.92 ± 0.53a	11.06 ± 0.15a	1.38 ± 0.01a	8.02 ± 0.05b
	10F	54.94 ± 1.38a	583.86 ± 6.18a	72.77 ± 0.05a	10.63 ± 0.23b	1.33 ± 0.03b	8.02 ± 0.09ab
	15F	48.89 ± 1.48c	547.00 ± 3.91b	66.94 ± 1.34b	11.30 ± 0.15a	1.37 ± 0.02a	8.17 ± 0.12a
S2	1F	43.48 ± 0.66d	538.82 ± 2.56b	63.41 ± 1.09b	12.39 ± 0.23a	1.46 ± 0.01a	8.50 ± 0.18b
	5F	50.59 ± 0.14a	583.49 ± 3.47a	69.81 ± 0.19a	11.53 ± 0.07b	1.38 ± 0.01b	8.36 ± 0.05bc
	10F	45.49 ± 1.39c	530.85 ± 4.23c	59.95 ± 0.33c	11.78 ± 0.10b	1.32 ± 0.03c	8.85 ± 0.05a
	15F	47.63 ± 0.78b	522.80 ± 1.65d	64.09 ± 0.56b	10.98 ± 0.17c	1.35 ± 0.03bc	8.16 ± 0.10c
S3	1F	49.35 ± 0.59a	488.44 ± 1.27a	59.48 ± 0.08a	9.90 ± 0.12b	1.21 ± 0.01b	8.21 ± 0.02a
	5F	45.02 ± 0.08b	465.37 ± 3.60b	57.18 ± 0.26b	10.34 ± 0.07a	1.27 ± 0.01a	8.14 ± 0.06ab
	10F	43.94 ± 1.10bc	459.23 ± 7.76b	56.86 ± 1.05b	10.31 ± 0.25a	1.29 ± 0.03a	7.99 ± 0.07c
	15F	43.53 ± 0.50c	457.30 ± 12.32b	56.36 ± 0.39b	10.51 ± 0.27a	1.29 ± 0.02a	8.05 ± 0.09bc
S4	1F	49.15 ± 1.51a	496.14 ± 4.61a	63.17 ± 0.95a	9.97 ± 0.16d	1.29 ± 0.03c	7.86 ± 0.19ab
	5F	39.17 ± 0.36c	434.89 ± 2.55c	53.81 ± 0.59c	11.10 ± 0.06a	1.37 ± 0.02b	8.08 ± 0.05a
	10F	43.15 ± 0.56b	455.01 ± 12.99b	59.25 ± 0.10b	10.62 ± 0.08b	1.37 ± 0.02b	7.68 ± 0.22b
	15F	37.56 ± 0.26d	387.21 ± 5.52d	54.06 ± 0.46c	10.31 ± 0.20c	1.44 ± 0.02a	7.16 ± 0.05c

Note: The stoichiometric ratio presented in the table reflects the relationship between soil organic carbon and total nutrient content. The soil nutrient data for S1 through S4 were collected on April 15, 2023, April 26, 2023, May 6, 2023, and June 22, 2023, respectively, marking the conclusion of the four developmental periods. The distinct lowercase letters within the same column of the table signify significant differences among various fertilization treatments during the same period (*P* < 0.05). The values reported are expressed as the mean ± standard deviation.

### 3.6. Effect of fertilization on the absorption of mineral elements in blueberry

As illustrated in Table 5, significant differences in the uptake of mineral nutrients (N, P, K) by blueberries were observed across various developmental stages and fertilizer treatments. Nutrient uptake exhibited a proportional increase with fertilizer application rates, indicating a dose-dependent relationship. Blueberries demonstrated stage-specific nutrient requirements, reflecting both temporal variations for the same element and inter-element differences within the same stage. Notably, nitrogen (N) requirements consistently surpassed those of phosphorus (P) and potassium (K), with P exhibiting the lowest demand. Nutrient requirements progressively increased throughout development, peaking during the fruit expansion phase when N, P, and K uptake reached 2.95 to 10.55 times, 3.05 to 6.53 times, and 2.77 to 8.54 times those of the preceding stages respectively. Daily absorption rates during fruit expansion were 0.74 to 3.70 times (N), 0.91 to 3.43 times (P), and 0.69 to 4.44 times (K) higher than those of earlier stages.

**Table 5:** Effects of different fertilizer application rates at different stages on mineral element absorption and daily absorption of blueberry.

Treatment		N uptake(mg)	P uptake(mg)	K uptake(mg)	N daily uptake(mg)	P daily uptake(mg)	K daily uptake(mg)
Fertilizing season	Fertilizer amount						
S1	1F	77.16 ± 0.52d	30.08 ± 2.37d	47.33 ± 0.61d	3.67 ± 0.02d	1.43 ± 0.11d	2.25 ± 0.03d
	5F	388.61 ± 33.31c	49.38 ± 2.69c	98.56 ± 3.00c	18.51 ± 1.59c	2.35 ± 0.13c	4.69 ± 0.14c
	10F	859.07 ± 6.22b	64.52 ± 2.99b	188.64 ± 4.61b	40.91 ± 0.30b	3.07 ± 0.14b	8.98 ± 0.22b
	15F	1224.41 ± 29.08a	72.56 ± 2.45a	219.80 ± 12.40a	58.31 ± 1.38a	3.46 ± 0.12a	10.47 ± 0.59a
S2	1F	79.14 ± 2.06d	14.41 ± 2.32d	62.19 ± 5.30d	7.91 ± 0.21d	1.44 ± 0.23d	6.22 ± 0.53d
	5F	421.00 ± 13.97c	33.02 ± 2.59c	118.57 ± 3.15c	42.10 ± 1.40c	3.30 ± 0.26c	11.86 ± 0.32c
	10F	770.61 ± 21.47b	70.68 ± 1.59b	172.14 ± 3.98b	77.06 ± 2.15b	7.07 ± 0.16b	17.21 ± 0.40b
	15F	995.42 ± 78.31a	80.88 ± 1.50a	217.51 ± 5.40a	99.54 ± 7.83a	8.09 ± 0.15a	21.75 ± 0.54a
S3	1F	160.03 ± 8.78d	21.97 ± 1.41d	85.60 ± 1.14d	16.00 ± 0.88d	2.20 ± 0.14d	8.56 ± 0.11d
	5F	314.17 ± 22.66c	51.06 ± 6.00c	109.90 ± 4.46c	31.42 ± 2.27c	5.11 ± 0.60c	10.99 ± 0.45c
	10F	714.49 ± 27.54b	67.38 ± 2.00b	235.12 ± 11.40b	71.45 ± 2.75b	6.74 ± 0.20b	23.51 ± 1.14b
	15F	773.73 ± 22.02a	109.69 ± 2.69a	395.89 ± 10.83a	77.37 ± 2.20a	10.97 ± 0.27a	39.59 ± 1.08a
S4	1F	471.40 ± 17.00d	91.72 ± 0.54d	237.11 ± 2.39d	11.78 ± 0.42d	2.29 ± 0.01d	5.93 ± 0.06d
	5F	2736.22 ± 11.58c	186.67 ± 9.88c	694.62 ± 2.28c	68.41 ± 0.29c	4.67 ± 0.25c	17.37 ± 0.06c
	10F	5323.94 ± 20.04b	312.89 ± 23.17b	1348.67 ± 32.43b	133.10 ± 0.50b	7.82 ± 0.58b	33.72 ± 0.81b
	15F	8159.06 ± 20.92a	474.03 ± 18.39a	1857.65 ± 56.90a	203.98 ± 0.52a	11.85 ± 0.46a	46.44 ± 1.42a

Note: The absorption of mineral elements presented in the table is calculated based on the content of available nutrients. The different lowercase letters within the same column signify significant differences among various fertilization treatments during the same period (*P* < 0.05), with the values representing the mean ± standard deviation.

## 4. Discussion

### 4.1. Response of soil nutrient content and stoichiometric ratio to fertilization amount

Fertilization increases exogenous nutrient inputs, enhances soil structure and fertility, and accelerates nutrient cycling [29]. In this study, following independent fertilization at four developmental stages, the total and available nitrogen (N), phosphorus (P), and potassium (K) contents in the 5F to 15F treatments were significantly higher than those observed prior to fertilization. This indicates that fertilization during the four developmental stages of blueberry effectively improves soil nutrient content. These findings align with previous studies [30–33]. During the fruit expansion phase, the availability of N, P, and K followed the trend 15F > 10F > 5F > 1F, suggesting dose-dependent relationships. Higher fertilizer rates provided more labile nutrients for fruit development, corroborating Liu Shuangfeng’s report [33] that greater available P increases occur with higher P application rates. Although soil organic carbon (SOC) and soil organic matter (SOM) fluctuated with varying fertilizer rates and application frequencies during fruit expansion, the 1F to 15F treatments maintained relatively high levels. This suggests that a stoichiometry-based fertilization approach with a 10-day interval replenishes nutrients absorbed by plants and supports SOC/SOM retention, consistent with prior studies [34–36].

Soil stoichiometric ratios serve as indicators of nutrient status, fertility, and organic matter decomposition. The C:N, C:P, and C:K ratios provide insights into mineralization and immobilization processes, where lower ratios signify enhanced mineralization and nutrient release [37]. The reported average stoichiometric ratios for Chinese soils are 11.9 for C:N, 61 for C:P, and 5.2 for N:P [38]. Prescott [39] identified that N mineralization diminishes when the C:N ratio in forest soils exceeds 35. Additionally, Jia Yu [40] noted that net organic phosphorus mineralization occurs at C:P ratios below 200, while phosphorus immobilization is observed at C:P ratios above 300. In our study, post-fertilization ratios of C:N (>35), C:P (>300), and N:P (>5.2) exhibited significant variation, likely attributable to substrate texture and/or insufficient available nutrients. However, it is important to note that this study primarily focused on the stoichiometric ratios of blueberry leaves under different fertilization treatments. The soil stoichiometric characteristics, including C:N, C:P, C:K, N:P, N:K, and K:P ratios, showed minimal fluctuations across the study periods. This stability suggests that the nitrogen, phosphorus, and potassium ratios can enhance the soil’s capacity to retain fertilizers and supply nutrients, potentially mitigating the risk of blueberry plants experiencing single salt toxicity.

### 4.2. Effect of fertilization on the absorption of mineral elements in blueberry

The uptake of plant nutrients from the soil is a continuous process throughout growth, influenced by fertilizer application rates, stage-specific demands, and complex synergistic and antagonistic interactions among nutrients, as well as between nutrients and soil [41]. Sun Mei [42] investigated nutrient uptake in Muscat Hamburg grapes under varying nutrient solution concentrations, revealing that the absorption of nitrogen (N), phosphorus (P), and potassium (K) followed a dose-dependent pattern of 1.5× > 1× > 0.5× across the flowering, young fruit, expansion, coloring, and ripening stages. This finding aligns with our results, which indicate increased N, P, and K uptake, along with daily absorption rates in blueberries, corresponding to higher fertilizer inputs during all four growth stages. Throughout these stages, blueberries consistently exhibited the highest N uptake, corroborating previous reports [43] that highlight the critical nitrogen demands during development. The pronounced peak in nutrient absorption during the fruit expansion phase corresponds to the rapid growth of the fruit, which necessitates substantial nutrient accumulation. Under the 15F treatment, maximum uptake values of 8.16 g N, 0.47 g P, and 1.86 g K were recorded, translating to 38.50 g of ammonium sulfate, 1.93 g of superphosphate, and 4.15 g of potassium sulfate. This underscores the necessity for timely NPK supplementation during the fruit expansion phase. Notably, the N, P, and K uptake in the 5F to 15F treatments generally lagged behind fertilizer inputs, suggesting either low nutrient use efficiency or limited bioavailability of soil nutrients. Future studies could investigate the potential of organic fertilizer amendments [44] or the inoculation of dark septate endophytes (DSE) [45] to enhance nutrient absorption efficiency.

## 5. Conclusion

The application of varying fertilizer rates based on leaf stoichiometric ratios during the four developmental stages of blueberry cultivation effectively increased the soil’s available nitrogen, phosphorus, and potassium contents while maintaining stoichiometric stability. The available nutrient contents exhibited dose-dependent relationships with fertilizer inputs across all four stages. Blueberry nutrient uptake increased proportionally with fertilizer rates, demonstrating a clear dose-dependent relationship for mineral nutrient absorption. Notably, nitrogen uptake consistently surpassed that of phosphorus and potassium throughout all stages, with the highest absorption of nitrogen, phosphorus, and potassium occurring during the fruit expansion stage. These findings underscore the significance of stage-specific fertilization management, emphasizing the prioritization of nitrogen supply while ensuring timely supplementation of phosphorus and potassium. Furthermore, balancing soil organic matter accumulation and mineralization is critical for maintaining nutrient availability and promoting soil health.

## Supporting information

S1 File Dataset (Figs 2–5). (XLSX)

## References

[1] Elser JJ, Sterner RW, Gorokhova E, Fagan WF, Markow TA, Cotner JB, et al. Biological stoichiometry from genes to ecosystems. Ecol Lett. 2000;3(6):540–50. doi: 10.1111/j.1461-0248.2000.00185.x

[2] Zechmeister-Boltenstern S, Keiblinger KM, Mooshammer M, Peñuelas J, Richter A, Sardans J, et al. The application of ecological stoichiometry to plant–microbial–soil organic matter transformations. Ecol Monogr. 2015;85(2):133–55. doi: 10.1890/14-0777.1

[3] Wang L, Zhang G, Zhu P, Xing S, Wang C. Soil C, N and P contents and their stoichiometry as affected by typical plant communities on steep gully slopes of the Loess Plateau, China. CATENA. 2022;208:105740. doi: 10.1016/j.catena.2021.105740

[4] Dong X, Xu DH, Han CX, Hai L, Liu Y, Zhang JB. Responses of ecological stoichiometric characteristics in organs of Ammopiptanthus mongolicus to heterogeneous habitats. Acta Ecologica Sinica. 2024;44(2):858–69. doi: 10.20103/j.stxb.202209282749

[5] Song LQ. The regulatory effects of controlled-release fertilizer and melatonin application on wheat yield and soil properties under FACE-O3 conditions. Yangzhou: Yangzhou University; 2024.

[6] Wang X, Zhang H, Cao D, Wu C, Wang X, Wei L, et al. Microbial carbon and phosphorus metabolism regulated by C:N:P stoichiometry stimulates organic carbon accumulation in agricultural soils. Soil Tillage Res. 2024;242:106152. doi: 10.1016/j.still.2024.106152

[7] Wang S, Wang W, Rong S, Liu G, Li Y, Wang X, et al. Key factors and mechanisms of microplastics’ effects on soil nitrogen transformation: a review. Soil Environ Health. 2024;2(4):100101. doi: 10.1016/j.seh.2024.100101

[8] Fan Y-R, Yu B-B, Xia S-P, Zhu Z-Q, Fan Y-C, Liu X-Y, et al. Responses of the contents and stoichiometry of soil carbon, nitrogen, and phosphorus to elevated atmospheric CO2 concentration and warming in paddy field. Ying Yong Sheng Tai Xue Bao. 2024;35(12):3409–18. doi: 10.13287/j.1001-9332.202412.013 40383892

[9] Maathuis FJM. Physiological functions of mineral macronutrients. Curr Opin Plant Biol. 2009;12(3):250–8. doi: 10.1016/j.pbi.2009.04.003 19473870

[10] Wu H, Dong SJ, Wang WH. Coupling relationship between physical and chemical properties of soil within pine-oak mixed forest in Qinling mountains. J Central South Univ For Technol. 2020;40(10):117–26. doi: 10.14067/j.cnki.1673-923x.2020.10.014

[11] Liu S-X, An H, Zhang X-W, Xing B-B, Wen Z-L, Wang B. Effects of nitrogen and phosphorus addition on soil nutrient content and stoichiometry in desert grassland. Huan Jing Ke Xue. 2023;44(5):2724–34. doi: 10.13227/j.hjkx.202205072 37177945

[12] Zhang Y, Li HT, Zhang H, Huang BB, Liu CH, Jiang ZK, et al. Soil C : N : P stoichiometry and its relationship with the soil physicochemical properties of different aged Chinese fir (Cunninghamia lanceolata) plantations. Acta Ecologica Sinica. 2019;39(7):2520–31.

[13] Hu Q, Shun R, Yang C, Zhang YY, Fu QM, Wu ZX. Soil ecological stoichiometric characteristics and influencing factors of rubber forest in Hainan Province. J For Environ. 2024;44(06):600–7. doi: 10.13324/j.cnki.jfcf.2024.06.005

[14] Liu R, Wang D. Soil C, N, P and K stoichiometry affected by vegetation restoration patterns in the alpine region of the Loess Plateau, Northwest China. PLoS One. 2020;15(11):e0241859. doi: 10.1371/journal.pone.0241859 33151996 PMC7644019

[15] Li YD. Blueberry (blueberry) cultivation and processing utilization. Changchun: Jilin Science and Technology Publishing House; 2001.

[16] Han PX, Zhang P, Fong XJ, Huang XJ, Li MM, Yang FW. Nutritional and health functions of blueberry and its development and utilization. Sci Technol Food Industry. 2015;36(6):370–5. doi: 10.13386/j.issn1002-0306.2015.06.072

[17] Lin B-W, Gong C-C, Song H-F, Cui Y-Y. Effects of anthocyanins on the prevention and treatment of cancer. Br J Pharmacol. 2017;174(11):1226–43. doi: 10.1111/bph.13627 27646173 PMC5429338

[18] Tena N, Martín J, Asuero AG. State of the art of anthocyanins: antioxidant activity, sources, bioavailability, and therapeutic effect in human health. Antioxidants (Basel). 2020;9(5):451. doi: 10.3390/antiox9050451 32456252 PMC7278599

[19] Krga I, Milenkovic D. Anthocyanins: from sources and bioavailability to cardiovascular-health benefits and molecular mechanisms of action. J Agric Food Chem. 2019;67(7):1771–83. doi: 10.1021/acs.jafc.8b06737 30698008

[20] Mattioli R, Francioso A, Mosca L, Silva P. Anthocyanins: a comprehensive review of their chemical properties and health effects on cardiovascular and neurodegenerative diseases. Molecules. 2020;25(17):3809. doi: 10.3390/molecules25173809 32825684 PMC7504512

[21] Bai X, Zhou L, Zhou L, Cang S, Liu Y, Liu R, et al. The research progress of extraction, purification and analysis methods of phenolic compounds from blueberry: a comprehensive review. Molecules. 2023;28(8):3610. doi: 10.3390/molecules28083610 37110844 PMC10140916

[22] Yang MH, Xie DH, Zhang X, Kuang HO, Li X, Tan X, et al. Analysis of major quality characteristics and volatile components of blueberry honey. Sci Technol Food Industry. 2022;43(14):336–44. doi: 10.13386/j.issn1002-0306.2021100197

[23] Tang Y. Effects of high ammonium stress on soil properties, growth and physiology of blueberry seedlings. Dalian: Dalian University of Technology; 2018.

[24] Gerard Krewer JR. Fertilizing Highbush Blueberries in Pine Bark Beds. University of Georgia; 2012. pp. 1–7.

[25] Liu HD, Song Y, Zhang HJ. Analysis on nutrient element contents of soils and leaves in blueberry orchards. South China Fruits. 2016;45(6):32–8. doi: 10.13938/j.issn.1007-1431.20160250

[26] Zheng WJ, Bao WK, Gu B, He X, Leng L. Carbon concentration and its characteristics in terrestrial higher plants. Chinese J Ecol. 2007;(3):307–13.

[27] Zeiger LTE. Plant Physiology. 5 ed. Bei Jing: Science Press; 2015.

[28] Wu WH. Plant physiology. Beijing: Science Press; 2018.

[29] Yu G, Chen F, Lu X, Teng MH, Tian X, Luo YL. Effects of different fertilization on soil nutrients and enzyme activities in young citrus orchards. Jiangsu Agric Sci. 2023;51(20):218–23. doi: 10.15889/j.issn.1002-1302.2023.20.031

[30] Li R, Chang Y, Hu T, Jiang X, Liang G, Lu Z, et al. Effects of Different Fertilization Treatments on Soil, Leaf Nutrient and Fruit Quality of Citrus grandis var. longanyou. World J Eng Technol. 2017;05(02):1–14. doi: 10.4236/wjet.2017.52b001

[31] Li F, Hao BG, Yu JL, Wu ME, Fong X, Li Y. Effect of formulated fertilizer application on N, P, K and stoichiometry ratios of leaves, branches, roots and soils of Larix principis-rupprechtii. J Central South Univ For Technol. 2023;43(12):126–36. doi: 10.14067/j.cnki.1673-923x.2023.12.012

[32] Li SY. Effects of nitrogen, phosphorus and potassium combined application on the quality, yield and soil microecology of Bupleurum chinense DC. Changchun: Jilin Agricultural University; 2023.

[33] Liu SF, Yang DZ, Wang F, Xu JL. Meta-analysis of the effect of fertilizer application on phosphorus forms in agricultural soils in China. Soil Fertilizer Sci China. 2024;8:43–51.

[34] Ladha JK, Reddy CK, Padre AT, van Kessel C. Role of nitrogen fertilization in sustaining organic matter in cultivated soils. J Environ Qual. 2011;40(6):1756–66. doi: 10.2134/jeq2011.0064 22031558

[35] Ashraf MN, Hu C, Wu L, Duan Y, Zhang W, Aziz T, et al. Soil and microbial biomass stoichiometry regulate soil organic carbon and nitrogen mineralization in rice-wheat rotation subjected to long-term fertilization. J Soils Sediments. 2020;20(8):3103–13. doi: 10.1007/s11368-020-02642-y

[36] Liu Y, Li C, Cai G, Sauheitl L, Xiao M, Shibistova O, et al. Meta-analysis on the effects of types and levels of N, P, and K fertilization on organic carbon in cropland soils. Geoderma. 2023;437:116580. doi: 10.1016/j.geoderma.2023.116580

[37] Wang SQ, Yu GR. Ecological stoichiometry characteristics of ecosystem carbon, nitrogen and phosphorus elements. Acta Ecologica Sinica. 2008;8:3937–47.

[38] Tian H, Chen G, Zhang C, Melillo JM, Hall CAS. Pattern and variation of C:N:P ratios in China’s soils: a synthesis of observational data. Biogeochemistry. 2009;98(1–3):139–51. doi: 10.1007/s10533-009-9382-0

[39] Prescott CE, Chappell HN, Vesterdal L. Nitrogen turnover in forest floors of coastal douglas-fir at sites differing in soil nitrogen capital. Ecology. 2000;81(7):1878–86. doi: 10.1890/0012-9658(2000)081[1878:ntiffo]2.0.co;2

[40] Yu J, Bingcheng X, Fengmin L, Xiaoling W. Availability and contributions of soil phosphorus to forage production of seeded alfalfa in semiarid Loess Plateau. Acta Ecologica Sinica. 2007;27(1):42–7. doi: 10.1016/s1872-2032(07)60009-2

[41] Hu Y. Researches on diagnosis and recommendation integrated system (DRIS) indexes of Juglans regia Xinwen185’ in Wushi County, Xinjiang. Urumqi: Xinjiang Agricultural University. 2015.

[42] Shun M. Study on nutrition and water absorption of Muscat Hamburg grapevine. Yinchuan: Ningxia University. 2017.

[43] Komosa A, Komosa A, Roszyk J, Mieloch M. Content of nutrients in soils of highbush blueberry (Vaccinium corymbosum L.) plantations in Poland in a long-term study. J Elem. 2017;(4/2017). doi: 10.5601/jelem.2016.21.4.1329

[44] Li ZY. Effect of bio-organic fertilizer on soil microbial properties and nutrients in blueberry root zone. Res Soil Water Conserv. 2017;24(2):36–42. doi: 10.13869/j.cnki.rswc.2017.02.006

[45] Tan Y. Study on the mechanism of DSE fungi promoting blueberry phosphorus absorption. Guiyang: Guizhou University; 2023.

